# 5,5′-Diphenyl-2,2′-[butane-1,4-diylbis(sulfanedi­yl)]bis­(1,3,4-oxadiazole)

**DOI:** 10.1107/S1600536810042315

**Published:** 2010-10-23

**Authors:** Wei Wang, Hong Qiu, Yan Gao, Hong-Guo Yao, Ming Ji

**Affiliations:** a School of Perfume and Aroma Technology, Shanghai Institute of Technology, Shanghai 200235, People’s Republic of China; b School of Chemical Engineering, University of Science and Technology LiaoNing, Anshan 114051, People’s Republic of China; c Liaoyang Supervision and Examination Station of Product Quality, Liaoning Liaoyang 111000, People’s Republic of China

## Abstract

The complete mol­ecule of the title compound, C_20_H_18_N_4_O_2_S_2_, is generated by crystallographic inversion symmetry. The benzene ring is almost coplanar with the oxadiazole ring [dihedral angle = 7.2 (2)°].

## Related literature

Functionalized 1,3,4-oxadiazole derivatives are of inter­est because of their biological activity and their wide applications in medicine, coordination chemistry and their use as organic electroluminescent (EL) devices, since these compounds possess good electron-accepting properties, see: Bentiss *et al.* (2000 ▶); Hughes & Bryce (2005 ▶); Navidpour *et al.* (2006 ▶).
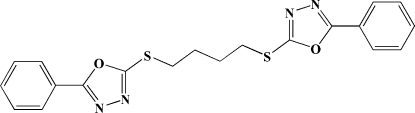


## Experimental

### 

#### Crystal data

C_20_H_18_N_4_O_2_S_2_
*M* *_r_* = 410.50
Monoclinic, 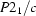

*a* = 12.202 (2) Å
*b* = 5.9317 (12) Å
*c* = 13.518 (3) Å
β = 104.04 (3)°
*V* = 949.2 (3) Å^3^
*Z* = 2
Mo *K*α radiation
μ = 0.31 mm^−1^
*T* = 113 K
0.20 × 0.18 × 0.12 mm

#### Data collection

Rigaku Saturn CCD area-detector diffractometer
Absorption correction: multi-scan (*CrystalClear*; Rigaku/MSC, 2005 ▶) *T*_min_ = 0.942, *T*_max_ = 0.964
7030 measured reflections
1661 independent reflections
1323 reflections with *I* > 2σ(*I*)
*R*_int_ = 0.053

#### Refinement

*R*[*F* ^2^ > 2σ(*F* ^2^)] = 0.040
*wR*(*F* ^2^) = 0.128
*S* = 1.10
1661 reflections
128 parameters
H-atom parameters constrained
Δρ_max_ = 0.28 e Å^−3^
Δρ_min_ = −0.33 e Å^−3^

### 

Data collection: *CrystalClear* (Rigaku/MSC, 2005 ▶); cell refinement: *CrystalClear*; data reduction: *CrystalClear*; program(s) used to solve structure: *SHELXS97* (Sheldrick, 2008 ▶); program(s) used to refine structure: *SHELXL97* (Sheldrick, 2008 ▶); molecular graphics: *SHELXTL* (Sheldrick, 2008 ▶); software used to prepare material for publication: *SHELXTL*.

## Supplementary Material

Crystal structure: contains datablocks global, I. DOI: 10.1107/S1600536810042315/zs2072sup1.cif

Structure factors: contains datablocks I. DOI: 10.1107/S1600536810042315/zs2072Isup2.hkl

Additional supplementary materials:  crystallographic information; 3D view; checkCIF report

## Figures and Tables

**Fig. 1.**
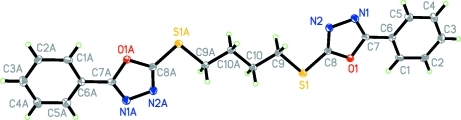
A view of the molecule of (I) showing the atom-labelling scheme. Atoms of the inversion-related atoms are indicated by symmetry code 'A' (-x + 1,-y + 3, -z + 1). Displacement ellipsoids are drawn at the 35% probability level.

## supplementary crystallographic information

### Comment

Functionalized 1,3,4-oxadiazole derivatives are of interest because of their biological activity and their wide applications in medicine, coordination chemistry and their use as organic electroluminescent (EL) devices, since these compounds possess good electron-accepting properties (Bentiss *et al.*, 2000; Navidpour *et al.*, 2006; Hughes & Bryce, 2005). We report here the synthesis and crystal structure of the title compound, C_20_H_18_N_4_O_2_S_2_ (I). In the structure of the title compound the molecule has an inversion centre at the mid-point of the central C10—C10^i^ bond (symmetry code for (i): -*x* + 1,-*y* + 3, -*z* + 1), the asymmetric unit containing half a molecule (Fig. 1). The mean plane of the oxadiazole ring is almost coplanar with the mean plane of the attached benzene ring [dihedral angle 7.2 (2)°]. As a result of π-π conjugation, the C*_sp_*^2^-S bond [S1—C8 = 1.729 (2) Å] is significantly shorter than the C*_sp_*^3^-S bond [S1—C9 = 1.818 (2) Å].

### Experimental

A suspension of 5-phenyl-1,3,4-oxadiazole-2-thiol (2.0 mmol) and 1,4-dibromobutane (1.0 mmol) in ethanol (10 ml) was stirred at room temperature. The reaction progress was monitored *via* TLC. The resulting precipitate was filtered off, washed with cold ethanol, dried and purified to give the title compound as a light yellow solid in 93% yield. Crystals of (I) suitable for single-crystal X-ray analysis were grown by slow evaporation of a solution in chloroform-ethanol (1:1).

### Refinement

All H atoms were positioned geometrically (C—H = 0.95–0.99 Å) and allowed to ride on their parent atoms, with *U*_iso_(H) = 1.2*U*_eq_(C).

### Crystal data

**Table:**

C_20_H_18_N_4_O_2_S_2_	*F*(000) = 428
*M**_r_* = 410.50	*D*_x_ = 1.436 Mg m^−^^3^
Monoclinic, *P*2_1_/*c*	Mo *K*α radiation, λ = 0.71073 Å
Hall symbol: -P 2ybc	Cell parameters from 3017 reflections
*a* = 12.202 (2) Å	θ = 1.7–27.9°
*b* = 5.9317 (12) Å	µ = 0.31 mm^−^^1^
*c* = 13.518 (3) Å	*T* = 113 K
β = 104.04 (3)°	Prism, colorless
*V* = 949.2 (3) Å^3^	0.20 × 0.18 × 0.12 mm
*Z* = 2	

### Data collection

**Table:**

Rigaku Saturn CCD area-detector diffractometer	1661 independent reflections
Radiation source: rotating anode	1323 reflections with *I* > 2σ(*I*)
confocal	*R*_int_ = 0.053
Detector resolution: 7.31 pixels mm^-1^	θ_max_ = 25.0°, θ_min_ = 1.7°
φ and ω scans	*h* = −12→14
Absorption correction: multi-scan (*CrystalClear*; Rigaku/MSC, 2005)	*k* = −7→6
*T*_min_ = 0.942, *T*_max_ = 0.964	*l* = −16→16
7030 measured reflections	

### Refinement

**Table:**

Refinement on *F*^2^	Secondary atom site location: difference Fourier map
Least-squares matrix: full	Hydrogen site location: inferred from neighbouring sites
*R*[*F*^2^ > 2σ(*F*^2^)] = 0.040	H-atom parameters constrained
*wR*(*F*^2^) = 0.128	*w* = 1/[σ^2^(*F*_o_^2^) + (0.0778*P*)^2^] where *P* = (*F*_o_^2^ + 2*F*_c_^2^)/3
*S* = 1.10	(Δ/σ)_max_ < 0.001
1661 reflections	Δρ_max_ = 0.28 e Å^−^^3^
128 parameters	Δρ_min_ = −0.33 e Å^−^^3^
0 restraints	Extinction correction: *SHELXL97* (Sheldrick, 2008), Fc^*^=kFc[1+0.001xFc^2^λ^3^/sin(2θ)]^-1/4^
Primary atom site location: structure-invariant direct methods	Extinction coefficient: 0.067 (10)

### Special details

**Table:**

Geometry. All e.s.d.'s (except the e.s.d. in the dihedral angle between two l.s. planes) are estimated using the full covariance matrix. The cell e.s.d.'s are taken into account individually in the estimation of e.s.d.'s in distances, angles and torsion angles; correlations between e.s.d.'s in cell parameters are only used when they are defined by crystal symmetry. An approximate (isotropic) treatment of cell e.s.d.'s is used for estimating e.s.d.'s involving l.s. planes.
Refinement. Refinement of *F*^2^ against ALL reflections. The weighted *R*-factor *wR* and goodness of fit *S* are based on *F*^2^, conventional *R*-factors *R* are based on *F*, with *F* set to zero for negative *F*^2^. The threshold expression of *F*^2^ > σ(*F*^2^) is used only for calculating *R*-factors(gt) *etc*. and is not relevant to the choice of reflections for refinement. *R*-factors based on *F*^2^ are statistically about twice as large as those based on *F*, and *R*- factors based on ALL data will be even larger.

### Fractional atomic coordinates and isotropic or equivalent isotropic displacement parameters (Å^2^)

**Table:**

	*x*	*y*	*z*	*U*_iso_*/*U*_eq_	
S1	0.43518 (5)	1.09305 (10)	0.65529 (4)	0.0256 (3)	
O1	0.30541 (12)	0.7504 (3)	0.68093 (12)	0.0210 (4)	
N1	0.19647 (17)	0.6825 (3)	0.52644 (14)	0.0265 (5)	
N2	0.26743 (17)	0.8685 (3)	0.51955 (14)	0.0242 (5)	
C1	0.2073 (2)	0.3923 (4)	0.77192 (18)	0.0278 (6)	
H1	0.2568	0.4939	0.8155	0.033*	
C2	0.1655 (2)	0.2040 (4)	0.81204 (19)	0.0292 (6)	
H2	0.1869	0.1762	0.8834	0.035*	
C3	0.0925 (2)	0.0563 (4)	0.7481 (2)	0.0291 (6)	
H3	0.0657	−0.0740	0.7757	0.035*	
C4	0.0589 (2)	0.0987 (4)	0.64463 (19)	0.0278 (6)	
H4	0.0071	−0.0001	0.6016	0.033*	
C5	0.1004 (2)	0.2843 (4)	0.60345 (18)	0.0258 (6)	
H5	0.0778	0.3123	0.5322	0.031*	
C6	0.17557 (19)	0.4300 (4)	0.66720 (17)	0.0185 (6)	
C7	0.22137 (19)	0.6207 (4)	0.62076 (17)	0.0193 (6)	
C8	0.3285 (2)	0.8996 (4)	0.61119 (17)	0.0214 (6)	
C9	0.4184 (2)	1.2482 (4)	0.53652 (18)	0.0235 (6)	
H9A	0.4274	1.1445	0.4817	0.028*	
H9B	0.3417	1.3145	0.5166	0.028*	
C10	0.5064 (2)	1.4340 (4)	0.55008 (17)	0.0216 (6)	
H10A	0.5829	1.3674	0.5705	0.026*	
H10B	0.4970	1.5378	0.6048	0.026*	

### Atomic displacement parameters (Å^2^)

**Table:**

	*U*^11^	*U*^22^	*U*^33^	*U*^12^	*U*^13^	*U*^23^
S1	0.0253 (4)	0.0301 (5)	0.0194 (4)	−0.0069 (3)	0.0015 (3)	0.0035 (2)
O1	0.0202 (9)	0.0232 (9)	0.0192 (9)	−0.0025 (7)	0.0038 (7)	0.0028 (6)
N1	0.0304 (12)	0.0260 (12)	0.0215 (11)	−0.0067 (10)	0.0035 (9)	−0.0009 (9)
N2	0.0293 (12)	0.0238 (11)	0.0191 (11)	−0.0046 (9)	0.0056 (9)	0.0011 (8)
C1	0.0193 (14)	0.0368 (16)	0.0239 (14)	−0.0036 (11)	−0.0014 (11)	0.0017 (10)
C2	0.0258 (14)	0.0363 (16)	0.0244 (13)	−0.0007 (12)	0.0039 (11)	0.0127 (11)
C3	0.0250 (14)	0.0234 (13)	0.0418 (16)	0.0021 (11)	0.0138 (12)	0.0038 (11)
C4	0.0325 (16)	0.0220 (14)	0.0316 (15)	−0.0059 (11)	0.0129 (12)	−0.0073 (10)
C5	0.0288 (14)	0.0296 (15)	0.0207 (13)	−0.0018 (11)	0.0093 (11)	−0.0062 (10)
C6	0.0180 (13)	0.0179 (13)	0.0214 (12)	0.0033 (10)	0.0084 (10)	−0.0013 (9)
C7	0.0160 (13)	0.0238 (14)	0.0171 (12)	0.0010 (10)	0.0020 (10)	−0.0039 (9)
C8	0.0202 (13)	0.0241 (14)	0.0196 (12)	0.0000 (10)	0.0045 (10)	0.0019 (9)
C9	0.0249 (13)	0.0244 (13)	0.0199 (12)	−0.0010 (11)	0.0031 (10)	0.0054 (10)
C10	0.0225 (13)	0.0212 (13)	0.0199 (13)	0.0021 (10)	0.0031 (10)	−0.0018 (9)

### Geometric parameters (Å, °)

**Table:**

S1—C8	1.729 (2)	C3—H3	0.9500
S1—C9	1.818 (2)	C4—C5	1.384 (3)
O1—C8	1.371 (3)	C4—H4	0.9500
O1—C7	1.378 (3)	C5—C6	1.396 (3)
N1—C7	1.290 (3)	C5—H5	0.9500
N1—N2	1.419 (3)	C6—C7	1.468 (3)
N2—C8	1.295 (3)	C9—C10	1.518 (3)
C1—C2	1.391 (3)	C9—H9A	0.9900
C1—C6	1.392 (3)	C9—H9B	0.9900
C1—H1	0.9500	C10—C10^i^	1.539 (4)
C2—C3	1.390 (4)	C10—H10A	0.9900
C2—H2	0.9500	C10—H10B	0.9900
C3—C4	1.382 (3)		
			
C8—S1—C9	96.73 (11)	C1—C6—C7	121.2 (2)
C8—O1—C7	101.66 (17)	C5—C6—C7	118.3 (2)
C7—N1—N2	106.53 (18)	N1—C7—O1	112.81 (19)
C8—N2—N1	105.48 (18)	N1—C7—C6	128.2 (2)
C2—C1—C6	119.2 (2)	O1—C7—C6	118.9 (2)
C2—C1—H1	120.4	N2—C8—O1	113.5 (2)
C6—C1—H1	120.4	N2—C8—S1	129.42 (18)
C3—C2—C1	120.2 (2)	O1—C8—S1	117.02 (17)
C3—C2—H2	119.9	C10—C9—S1	109.62 (17)
C1—C2—H2	119.9	C10—C9—H9A	109.7
C4—C3—C2	120.2 (2)	S1—C9—H9A	109.7
C4—C3—H3	119.9	C10—C9—H9B	109.7
C2—C3—H3	119.9	S1—C9—H9B	109.7
C3—C4—C5	120.3 (2)	H9A—C9—H9B	108.2
C3—C4—H4	119.9	C9—C10—C10^i^	110.2 (2)
C5—C4—H4	119.9	C9—C10—H10A	109.6
C4—C5—C6	119.6 (2)	C10^i^—C10—H10A	109.6
C4—C5—H5	120.2	C9—C10—H10B	109.6
C6—C5—H5	120.2	C10^i^—C10—H10B	109.6
C1—C6—C5	120.5 (2)	H10A—C10—H10B	108.1
			
C7—N1—N2—C8	0.1 (2)	C1—C6—C7—N1	−176.7 (2)
C6—C1—C2—C3	−0.4 (4)	C5—C6—C7—N1	4.3 (4)
C1—C2—C3—C4	−1.5 (4)	C1—C6—C7—O1	6.5 (3)
C2—C3—C4—C5	2.0 (4)	C5—C6—C7—O1	−172.54 (19)
C3—C4—C5—C6	−0.6 (4)	N1—N2—C8—O1	−0.1 (3)
C2—C1—C6—C5	1.8 (3)	N1—N2—C8—S1	177.74 (17)
C2—C1—C6—C7	−177.2 (2)	C7—O1—C8—N2	0.0 (2)
C4—C5—C6—C1	−1.3 (3)	C7—O1—C8—S1	−178.11 (15)
C4—C5—C6—C7	177.7 (2)	C9—S1—C8—N2	6.4 (2)
N2—N1—C7—O1	−0.1 (2)	C9—S1—C8—O1	−175.89 (17)
N2—N1—C7—C6	−177.1 (2)	C8—S1—C9—C10	−178.33 (17)
C8—O1—C7—N1	0.1 (2)	S1—C9—C10—C10^i^	179.7 (2)
C8—O1—C7—C6	177.40 (19)		

Symmetry codes: (i) −*x*+1, −*y*+3, −*z*+1.
